# COVID-19 infection, admission and death and the impact of corticosteroids among people with rare autoimmune rheumatic disease during the second wave of COVID-19 in England: results from the RECORDER Project

**DOI:** 10.1093/rheumatology/kead150

**Published:** 2023-04-05

**Authors:** Megan Rutter, Peter C Lanyon, Matthew J Grainge, Richard Hubbard, Mary Bythell, Peter Stilwell, Jeanette Aston, Sean McPhail, Sarah Stevens, Fiona A Pearce

**Affiliations:** Department of Lifespan and Population Health, School of Medicine, University of Nottingham, Nottingham, UK; Department of Rheumatology, Nottingham University Hospitals NHS Trust, Nottingham, UK; National Congenital Anomaly and Rare Disease Registration Service, National Disease Registration Service, NHS Digital, Leeds, UK; Department of Lifespan and Population Health, School of Medicine, University of Nottingham, Nottingham, UK; Department of Rheumatology, Nottingham University Hospitals NHS Trust, Nottingham, UK; National Congenital Anomaly and Rare Disease Registration Service, National Disease Registration Service, NHS Digital, Leeds, UK; National Institute for Health Research (NIHR), Nottingham Biomedical Research Centre, Nottingham, UK; Department of Lifespan and Population Health, School of Medicine, University of Nottingham, Nottingham, UK; Department of Lifespan and Population Health, School of Medicine, University of Nottingham, Nottingham, UK; National Institute for Health Research (NIHR), Nottingham Biomedical Research Centre, Nottingham, UK; National Congenital Anomaly and Rare Disease Registration Service, National Disease Registration Service, NHS Digital, Leeds, UK; National Congenital Anomaly and Rare Disease Registration Service, National Disease Registration Service, NHS Digital, Leeds, UK; National Congenital Anomaly and Rare Disease Registration Service, National Disease Registration Service, NHS Digital, Leeds, UK; National Congenital Anomaly and Rare Disease Registration Service, National Disease Registration Service, NHS Digital, Leeds, UK; National Congenital Anomaly and Rare Disease Registration Service, National Disease Registration Service, NHS Digital, Leeds, UK; Department of Lifespan and Population Health, School of Medicine, University of Nottingham, Nottingham, UK; Department of Rheumatology, Nottingham University Hospitals NHS Trust, Nottingham, UK; National Congenital Anomaly and Rare Disease Registration Service, National Disease Registration Service, NHS Digital, Leeds, UK; National Institute for Health Research (NIHR), Nottingham Biomedical Research Centre, Nottingham, UK

**Keywords:** COVID-19, coronavirus, mortality, rare autoimmune rheumatic diseases, epidemiology, shielding, infection

## Abstract

**Objectives:**

To calculate the rates of COVID-19 infection and COVID-19-related death among people with rare autoimmune rheumatic diseases (RAIRD) during the second wave of the COVID-19 pandemic in England, and describe the impact of corticosteroids on outcomes.

**Methods:**

Hospital Episode Statistics data were used to identify people alive on 1 August 2020 with ICD-10 codes for RAIRD from the whole population of England. Linked national health records were used to calculate rates and rate ratios of COVID-19 infection and death up to 30 April 2021. Primary definition of COVID-19-related death was mention of COVID-19 on the death certificate. NHS Digital and Office for National Statistics general population data were used for comparison. The association between 30-day corticosteroid usage and COVID-19-related death, COVID-19-related hospital admissions and all-cause deaths was also described.

**Results:**

Of 168 330 people with RAIRD, 9961 (5.92%) had a positive COVID-19 PCR test. The age-standardized infection rate ratio between RAIRD and the general population was 0.99 (95% CI: 0.97, 1.00). 1342 (0.80%) people with RAIRD died with COVID-19 on their death certificate and the age–sex-standardized mortality rate for COVID-19-related death was 2.76 (95% CI: 2.63, 2.89) times higher than in the general population. There was a dose-dependent relationship between 30-day corticosteroid usage and COVID-19-related death. There was no increase in deaths due to other causes.

**Conclusions:**

During the second wave of COVID-19 in England, people with RAIRD had the same risk of COVID-19 infection but a 2.76-fold increased risk of COVID-19-related death compared with the general population, with corticosteroids associated with increased risk.

Rheumatology key messages

- People with RAIRD had 2.76 times the risk of COVID-19-related death than the general population.
- There was no evidence of increased risk of death from non-COVID-19-related causes.
- Corticosteroids were associated with increased risk of COVID-19-related death, in a dose-dependent fashion.

## Introduction

The research community responded remarkably to the challenge of the COVID-19 pandemic, rapidly describing outcomes in people with rare autoimmune rheumatic diseases (RAIRD) in order to guide clinical practice. However, previous studies have been small and/or relied on case series and physician-reported cases [1–5]. This allowed only internal comparisons within cohorts of people with rheumatic diseases and not comparison with the general population.

Our previous work used whole population data to show that people with RAIRD were at increased risk of death related to COVID-19 infection during the first wave of the COVID-19 pandemic (1 March–31 July 2020), when compared with the general population in England [6, 7].

There were concerns that more transmissible SARS-CoV-2 variants (including the Alpha variant) may have further increased the risk to those with RAIRD. This study therefore repeats methods used in our previous work, using linked national health records for the whole population of England, to calculate the rates of laboratory confirmed COVID-19 infection and COVID-19-related death among people with RAIRD during the second wave of the COVID-19 pandemic (1 August 2020–30 April 2021) and compares these rates with those in the general population. We also describe COVID-19-related hospital and intensive care unit (ICU) admission, underlying causes of death by category and COVID-19-related mortality stratified by RAIRD diagnosis.

Finally, as registry studies have highlighted an association between corticosteroids and adverse COVID-19 outcomes [1, 8–10], we examine the effect of corticosteroids on COVID-19-related death using national community prescriptions data. We also examine the effect of corticosteroid dose, to establish to what extent a relationship between corticosteroid and mortality is influenced by dose.

## Methods

### Background

This work is a product of the collaboration between the Registration of Complex Rare Diseases Exemplars in Rheumatology (RECORDER) project at the University of Nottingham, Nottingham University Hospitals NHS Trust and the National Congenital Anomaly and Rare Disease Registration Service (NCARDRS). NCARDRS, based within NHS Digital (NHSD), registers people with rare conditions in order to support high quality clinical practice and research, provide whole population epidemiological data and empower patients [11]. It has unique access to linked national datasets of electronic health records at patient-identifiable level for the whole population of England.

This study uses Hospital Episode Statistics (HES; which contains every episode of admitted patient care in NHS hospitals in England), COVID-19 PCR test results, Office for National Statistics (ONS) death certificate data and NHS prescriptions dispensed in the community.

**Figure 1.**
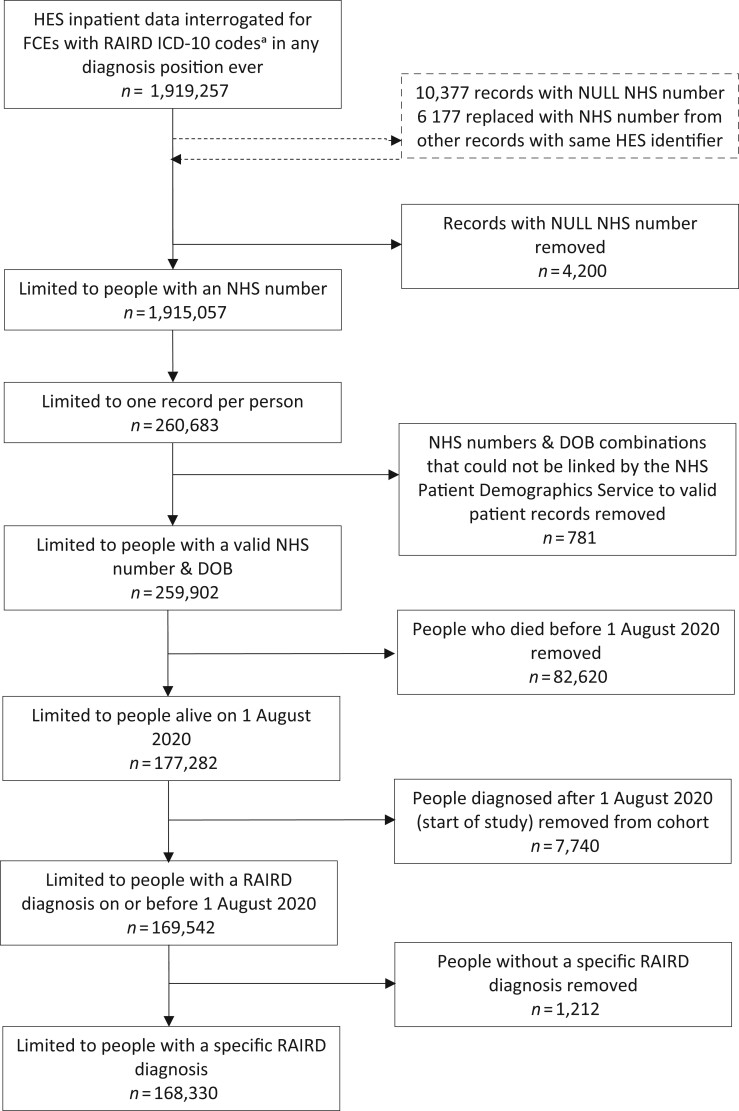
Data flow diagram showing identification of RAIRD cohort from HES data. ^a^RAIRD ICD-10 codes comprise: M313, M317, M301, M314, I776, M352, M315, M316, M321, M330, M332, M331, M339, M340, M341, M348, M349, M083, M084, M082, M080, M300, M308, J991, N085, N164, M328, M329, M609, G724, M608, M089. FCE: finished consultant episode; HES: hospital episode statistics; RAIRD: rare autoimmune rheumatic diseases

The RAIRD included in this study comprise ANCA-associated vasculitis, Behçet’s disease, giant cell arteritis, idiopathic inflammatory myopathies, juvenile inflammatory arthritis, scleroderma, systemic lupus erythematosus and Takayasu arteritis (ICD-10 code lists are shown in Fig. 1).

### Data validation

As reported in earlier papers, our previous work validating HES ICD-10 codes for RAIRD has shown high accuracy, with a positive predictive value of 84.7% [6] and prevalence estimates for individual RAIRD similar to reported population estimates.

### Study cohort

People with a diagnostic code ever for RAIRD in HES, resident in England, and alive on 1 August 2020 were included in the study. A data flow diagram is shown in Fig. 1. Vital status data from the NHS Personal Demographics Service were used to confirm whether people were alive, or where relevant, to confirm date of death [12].

Participants were grouped by RAIRD diagnosis, based on their most recent diagnostic code. Where the most recent code was non-specific, for example ‘Renal tubulo-interstitial disorder in systemic connective tissue disorder’, earlier, more specific diagnostic codes were used. Where only non-specific codes were available, participants were excluded, following the algorithm in Supplementary Data S1, available at *Rheumatology* online.

### COVID-19 infection

A population-level dataset of COVID-19 PCR test results, held in the Second Generation Surveillance System in UK Health Security Agency (UKHSA), was used to determine COVID-19 status. Positive tests among the RAIRD cohort were extracted, along with the date the laboratory reported the result. Demographics are described by pillar 1 (in-hospital) or pillar 2 (community) testing.

Laboratory confirmed COVID-19 infection rate from 1 August 2020 to 30 April 2021 was calculated, with the cohort of patients identified as having RAIRD used as the denominator population. Infection rate was age-standardized to the mid-year 2020 England population. Publicly available data from the government Coronavirus dashboard [13] were used to compare infection rates in the general population.

### Death certificate data

Cause of death data from death certificates (free text and ICD-10 coded) provided by the ONS were utilized. All cause of death fields were examined for ICD-10 codes specific to current COVID-19 infection (U07.1, U07.2). The free text was manually checked for keywords (‘cov’, ‘virus’ or ‘19’) which found that one death with a mention of COVID-19 was not coded with a relevant code. To align with the methodology used for the general population data, this was not included in the final analysis. We classified underlying all-cause death by category.

### All-cause and COVID-19-related mortality

The crude all-cause mortality rate from 1 August 2020 to 30 April 2021 was calculated, with the cohort of patients identified as having RAIRD used as the denominator population, along with the crude mortality rates for the two measures of COVID-19-related death.

We report two measures of COVID-19-related deaths. Our primary definition is death with any mention of COVID-19 on the death certificate as used by the ONS [14]. Our secondary definition is death within 28 days of a positive COVID-19 PCR test, as used by UKHSA [15].

Age-sex-standardized mortality rates per 100 000 in the population were calculated, standardized to the 2020 mid-year estimate for the England population using 5-year age bands. Age-standardized mortality rates (ASMRs) standardized to the 2013 European Standard Population (ESP) were also calculated. As the ESP is not disaggregated by sex and assumes equal numbers of males and females, and identical age distributions within sexes, this population was not used to calculate age–sex standardized rates.

The ONS provided general population data for all-cause deaths, and deaths with any mention of COVID-19 on the death certificate, over the same time period in England, split by sex and age band [16]. UKHSA provided comparable data for deaths within 28 days of a positive COVID-19 test (available to the public on request). These data were used to calculate the crude, age-standardized and age-specific mortality rates as a comparator. The 2020 mid-year estimate for the population of England was used as the denominator.

All-cause deaths in 2019 and 2020, deaths with any mention of COVID-19 on the death certificate and deaths within 28-days of a positive COVID-19 PCR test were plotted over time in order to allow direct comparison of the different measures, and to assess whether the peak in COVID-19-related deaths corresponded to a peak in all-cause mortality.

Underlying causes of death by ICD-10 category were extracted and categorized, e.g. cardiovascular disease, dementia. For comparison, data were extracted on all-cause death occurring during August–April 2016–2021 in people with a diagnostic code for RAIRD, to assess for an increase in deaths due to causes other than COVID-19 infection.

Age at death was calculated and compared with the time period described above, to examine for any impact of the COVID-19 pandemic.

### Stratification by disease

Poisson regression methods were used to analyse rates of COVID-19-related death, adjusted for age, sex and RAIRD diagnosis. The combined RAIRD cohort was used as the reference category. Incidence rate ratios for each diagnosis, with 95% confidence intervals, were displayed as a forest plot.

### Corticosteroid prescription data

A sub-analysis was performed in those testing positive for COVID-19. A national community prescriptions dataset [17, 18] was used to extract prescriptions for oral corticosteroids in this cohort.

Prednisolone equivalent dose (PED) in mean daily dose bands (0 mg, >0 mg–5 mg, >5 mg–10 mg, >10 mg–15 mg, >15 mg) was calculated for the 30-day period prior to COVID-19 infection. In those dying related to COVID-19 infection but without a positive PCR test (*n* = 102), date of infection was taken as date of death minus 10 days. Poisson regression was used to assess the effect of corticosteroid dose on COVID-19-related death, adjusted for age and sex. A *P*-value for trend was calculated.

Compared with the first wave of the pandemic, the proportion of COVID-19-related deaths occurring on the same day as the positive PCR test result, and therefore accruing zero person-time and being excluded from the Poisson analysis, increased from 2.9% (21/713) to 3.8% (51/1342). To assess whether excluding these deaths influenced the results, a sensitivity analysis was performed.

### Hospital and intensive care unit admissions

HES admitted patient care (APC) data on hospital and ICU admissions with an ICD-10 diagnostic code for COVID-19 were extracted. Duration and number of admissions, and basic and advanced respiratory support days on ICU are described.

### Ethics

This study was approved by the Camden and Kings Cross Research Ethics Committee, study reference 20/HRA/2076, on 18 June 2020.

The legal basis to access the data is covered by NCARDRS’s section 254 approval (sections 254(1) and 254(6) of the 2012 Health and Social Care Act), which includes a specific legal instruction to collect patient data without informed consent [19].

For quality assurance the data extraction and analysis were re-conducted by an independent analyst from the National Disease Registration Service.

### Patient and public involvement

This work has been developed with input from people with RAIRD. Following our initial findings of increased all-cause mortality during COVID-19 [6], we consulted with patients and patient charities to confirm priorities for future research and inform the communication and dissemination of our results. A plain English summary of this study is available as an online supplement.

### Data analysis

Cleaning, linkage and analysis of the data were performed in R version 4.1.0 (packages *tidyverse* [20], *janitor* [21], *survival* [22], *mfx* [23], *survminer* [24] and *meta* [25]).

## Results

### Cohort demographic data

The number of people meeting the inclusion criteria for this study was 168 330. Descriptive demographic data, including RAIRD diagnoses, are shown in Table 1. The median age of the population was 61.7 years (IQR 41.5–75.5) and 118 199 (70.2%) were female.

**Table 1.** Characteristics of the cohort of people with RAIRD alive 1 August 2020 (*n* = 168 330)

Characteristic	Value
Sex^a^, *n* (%)	
Female	118 199 (70.2)
Male	50 127 (29.8)
Age, mean (s.d.), years	
Total cohort	57.5 (22.5)
Female	58.3 (21.8)
Male	55.5 (24.0)
Age, median (IQR), years	
Total cohort	61.7 (41.5–75.5; 34.0)
Female	61.8 (43.0–75.7; 32.7)
Male	61.2 (36.4–74.9; 38.5)
Most recent diagnosis, *n* (%)	
Behçet's disease	4951 (2.9)
Dermatomyositis	2623 (1.6)
Eosinophilic granulomatosis with polyangiitis	2328 (1.4)
Giant cell arteritis	37 970 (22.6)
Granulomatosis with polyangiitis	6283 (3.7)
Arteritis, unspecified	16 502 (9.8)
Juvenile inflammatory arthritis	21 431 (12.7)
Juvenile myositis	505 (0.3)
Microscopic polyangiitis	1476 (0.9)
Polyarteritis nodosa	2517 (1.5)
Polymyositis	17 584 (10.4)
Scleroderma	11 530 (6.8)
Systemic lupus erythematosus	41 719 (24.8)
Takayasu arteritis	911 (0.5)

ICD-10 codes used: systemic lupus erythematosus: M321, M328, M329; GCA: M315, M316; JIA: M080, M082, M083, M084, M089; arteritis, unspecified: I776; polymyositis: G724, M332, M608, M609; scleroderma: M340, M341, M348, M349; granulomatosis with polyangiitis: M313; Behçet's disease: M352; dermatomyositis: M331, M339; eosinophilic granulomatosis with polyangiitis: M301; polyarteritis nodosa: M300, M308; microscopic polyangiitis: M317; Takayasu arteritis: M314; juvenile myositis: M330.

a Four patients had no sex recorded. IQR: interquartile range; RAIRD: rare autoimmune rheumatic diseases.

### COVID-19 infection

Between 1 August 2020 and 30 April 2021, 9961 (5.92%) of the RAIRD population had a positive COVID-19 PCR test, compared with 3 466 193 (6.13%) of the general population. Age-standardized to the England population, the infection rate per 100 000 person-years was 8073.6 (95% CI: 7936.3, 8210.9), similar to 8172.6 (95% CI: 8165.1, 8180.0) per 100 000 person-years in the general population (rate ratio 0.99 [95% CI: 0.97, 1.00], Table 2).

**Table 2.** PCR-proven COVID-19 infections in the RAIRD population compared with the whole population of England

	RAIRD (*n* = 168 330)	England (*n* = 56 550 138)
Infection rate, *n* (%)	9961 (5.92)	3466 193 (6.13)
Infection fatality rate—death certificate mention of COVID, *n* (%)	1342 (0.80)	82 361 (0.15)
Infection fatality rate—death within 28 days of COVID test, *n* (%)	1196 (0.71)	75 564 (0.13)
Age-standardized COVID-19 infection rate (95% CI)^a^, per 100 000 person-years	8073.6 (7936.3–8210.9)	8172.6 (8165.1–8180.0)
Positive COVID-19 PCR tests by pillar		
In-hospital testing, *n* (% total tests)	3093 (31.05)	—
Community testing, *n* (% total tests)	6868 (68.95)	—
Total, *n*	9961	3 466 193
Age of those in RAIRD cohort with a positive PCR test, mean or median (IQR), years		
In-hospital testing	68.7	73.9 (57.9–83.0; 25.1)
Community testing	49.5	50.3 (30.7–66.1; 35.4)
Combined	55.5	57.0 (37.0–75.2; 38.2)
Deaths in RAIRD cohort within 28 days of a positive PCR test, by pillar		
In-hospital testing, *n* (% deaths)	876 (73.2)	
Community testing, *n* (% deaths)	320 (26.8)	
All, *n*	1196	
Age of RAIRD cohort who died within 28 days of a positive PCR test, mean or median (IQR), years		
In-hospital testing	77.1	78.9 (71.1–85.3; 14.2)
Community testing	78.6	82.2 (71.0–88.0; 17.0)

In-hospital testing refers to ‘pillar 1’ tests and community testing refers to ‘pillar 2’ tests.

a For age-standardized COVID-19 infection rate, the rate ratio is 0.99 (95% CI: 0.97, 1.00). IQR: interquartile range; RAIRD: rare autoimmune rheumatic diseases.

Characteristics of those with a positive COVID-19 PCR test are shown in Table 2. A lower proportion of those testing positive had a hospital test (pillar 1; 31.05% of positive tests) than during the first wave (86.87% of positive tests).

### All-cause mortality

Between 1 August 2020 and 30 April 2021, 5822 (3.46%) people in the RAIRD cohort died of any cause (Table 3).

**Table 3.** Age-sex-standardized mortality rates for RAIRD from 1 August 2020 to 30 April 2021, compared with the general population of England

	Number of deaths	Number of people	Person-years	Crude mortality rate per 100 000 person years (95% CI)	RAIRD age–sex-standardized mortality rate^a^ (95% CI)	England age–sex-standardized mortality rate^a^ (95% CI)	Risk ratio for mortality rates (95% CI)
All-cause mortality							
All	5822	168 330	125 902	4611.6 (4509.0, 4714.2)	2277.9 (2227.3, 2328.6)	1007.2 (1004.6, 1009.8)	2.26 (2.21, 2.31)
Female	3730	118 199	88 406	4207.6 (4090.7, 4324.5)	2000.1 (1944.5, 2055.7)	978.7 (975.1, 982.3)	2.04 (1.99, 2.10)
Male	2092	50 127	37 492	5564.5 (5358.0, 5771.0)	2561.6 (2466.5, 2656.6)	1036.3 (1032.5, 1040.0)	2.47 (2.38, 2.56)
Death with any mention of COVID-19 on the death certificate							
All	1342	168 330	125 902	1063.0 (1013.7, 1112.2)	535.5 (510.7, 560.3)	194.2 (193.0, 195.3)	2.76 (2.63, 2.89)
Female	825	118 199	88 406	930.6 (875.6, 985.6)	444.7 (418.4, 471.0)	177.0 (175.5, 178.6)	2.51 (2.36, 2.66)
Male	517	50 127	37 492	1375.2 (1272.5, 1477.8)	628.2 (581.3, 675.1)	211.7 (210.0, 213.4)	2.97 (2.75, 3.19)
Death within 28 days of a positive COVID-19 test							
All	1196	168 330	125 902	947.3 (900.8, 993.8)	476.6 (453.2, 500.0)	178.2 (177.1, 179.3)	2.67 (2.54, 2.81)
Female	744	118 199	88 406	839.3 (787.0, 891.5)	400.9 (376.0, 425.8)	162.6 (161.1, 164.1)	2.47 (2.31, 2.62)
Male	452	50 127	37 492	1202.2 (1106.3, 1298.3)	553.8 (509.6, 598.0)	194.1 (192.4, 195.7)	2.85 (2.63, 3.08)

a Sex-specific rates are age-standardized only. Note that four people had no sex recorded. RAIRD: rare autoimmune rheumatic diseases.

### COVID-19-related mortality

Of the 5822 people who died, death certificate data were available for 5651 (97%); 1342 (0.80% RAIRD cohort) had COVID-19 mentioned on their death certificate, in any position. This compares to 82 361 (0.15%) of all those who died in the general population.

Of those with a positive COVID-19 PCR test, 1196/9961 (12.0%) died within 28 days of a positive test, compared with 75 564/3 466 193 (2.2%) of the general population. However, the RAIRD cohort were older than the general population of England.

The combined total of people with RAIRD dying with either COVID-19 mentioned on their death certificate or within 28 days of a positive COVID-19 test is reported in Supplementary Data S2, available at *Rheumatology* online.

### Age- and age–sex-standardized mortality rates

The age–sex-standardized mortality rate for all-cause death in RAIRD, standardized to the 2020 mid-year population of England, was 2277.9 (95% CI: 2227.3, 2328.6), compared with 1007.2 (95% CI: 1004.6, 1009.8) in the general population (rate ratio 2.26 [95% CI: 2.21, 2.31]). For deaths mentioning COVID-19 on the death certificate, the age–sex-standardized mortality rate was 535.5 (95% CI: 510.7, 560.3), compared with 194.2 (95% CI: 193.0, 195.3) in the general population (rate ratio 2.76 [95% CI: 2.63, 2.89]). For deaths within 28 days of a positive COVID-19 PCR test, the age–sex-standardized mortality rate in RAIRD was 476.6 (95% CI: 453.2, 500.0), compared with 178.2 (95% CI: 177.1, 179.3) in the general population (rate ratio 2.67 [95% CI: 2.54, 2.81]). These data are summarized in Table 3. The ASMR for all-cause death in RAIRD, adjusted to the 2013 European Standard Population is available in Supplementary Table S1, available at *Rheumatology* online.

### COVID-19-related deaths over time

A plot showing all-cause deaths, deaths with any mention of COVID-19 on the death certificate and deaths within 28 days of a positive COVID-19 PCR test over time is shown in Supplementary Fig. S1, available at *Rheumatology* online.

### All-cause death by category

Where death certificate data were available (*n* = 5651), underlying causes of death by category were extracted and are shown in Supplementary Table S2, available at *Rheumatology* online. Deaths attributed to cardiovascular disease were recorded in 1216 (21.6%), COVID-19 1194 (21.1%), malignancy 1020 (18.0%), respiratory 539 (9.5%), dementia 430 (7.6%), underlying RAIRD 193 (3.4%) and non-COVID-19 infection 55 (1.0%), with the remaining 1004 (17.8%) ascribed to other causes.

**Figure 2.**
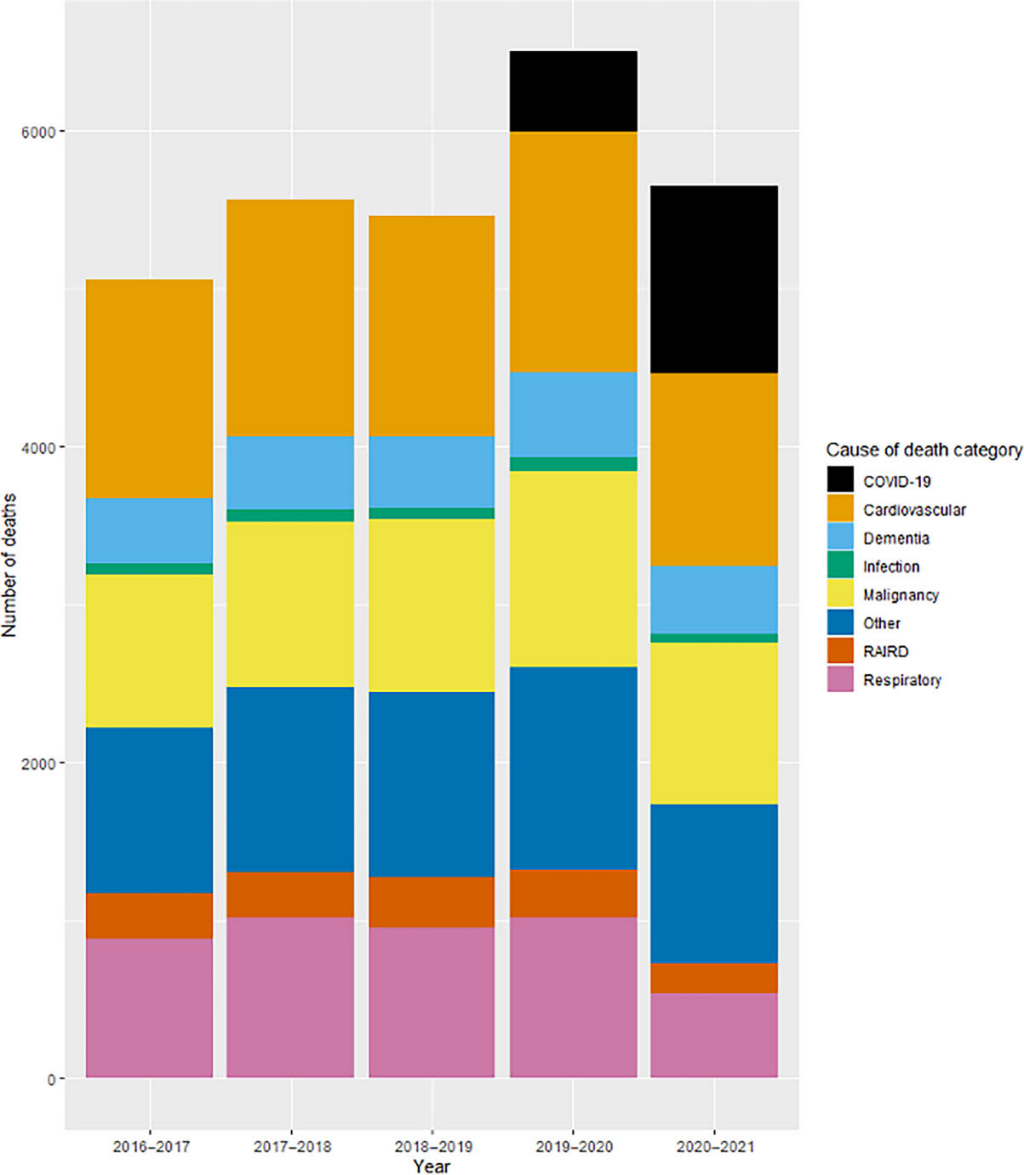
All cause death by category in people with RAIRD during months August–April in 2016–2021. Categories align to major ICD-10 code chapters, with the addition of diagnoses pertinent to the cohort (RAIRD, dementia). RAIRD: rare autoimmune rheumatic diseases

The comparison with all-cause death occurring during August–April 2016–2021 in people with a diagnostic code for RAIRD is shown in Fig. 2. As in the first wave of the pandemic, there remained no evidence of an increase in death from non-COVID-19-related causes.

### Age at death

There was no significant change in median age at death between August 2016 and April 2021 (median age ranged from 80.7 to 81.0), although, unlike wave one, age at death related to COVID-19 was slightly lower (median 80.2, IQR 70.8–86.6; Supplementary Table S3, available at *Rheumatology* online).

Age at death in people with RAIRD was compared between 2020–2021 and 2019–2020, categorized by underlying cause of death (Supplementary Fig. S2, available at *Rheumatology* online). There was no evidence of earlier age of death occurring in 2020 in certain cause of death categories, e.g. in deaths from cardiovascular disease.

### Stratification by disease

**Figure 3.**
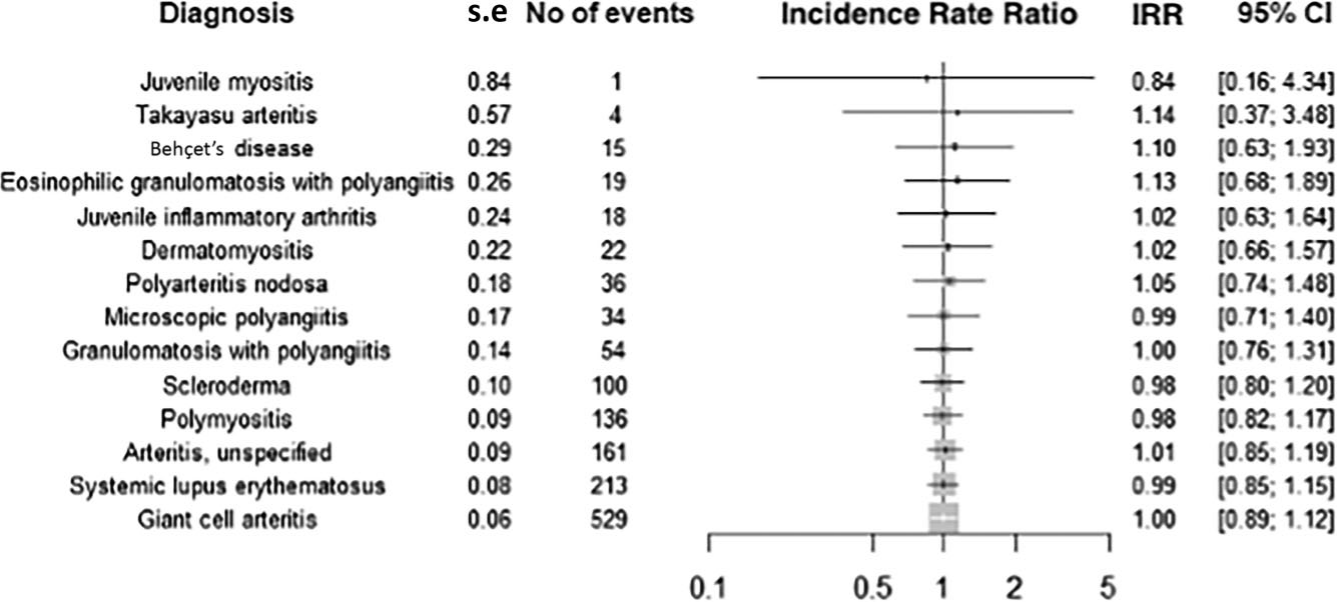
Forest plot showing incidence rate ratios with 95% CI for COVID-19-related death stratified by RAIRD diagnosis, with the combined RAIRD cohort used as the reference group. IRR: incidence rate ratio; RAIRD: rare autoimmune rheumatic diseases

Incidence rate ratios for COVID-19-related death stratified by RAIRD diagnosis are displayed in Fig. 3. The comparable ratios suggest a similar risk across the RAIRD cohort, regardless of diagnosis.

### Corticosteroid prescriptions data

Risk ratios for COVID-19-related death stratified by prednisolone equivalent dose (PED) are displayed in Table 4. There is a dose–response relationship with a 1.10 (95% CI: 1.08, 1.13) increase in risk of death for every additional 5 mg PED. Those taking >15 mg PED daily at the time of COVID-19 infection had 2.15 times (95% CI: 1.80, 2.56) higher COVID-19-related mortality than those not taking corticosteroids. The *P*-value for trend was <0.01. The results of the sensitivity analysis are shown in Supplementary Table S4, available at *Rheumatology* online; there was no material difference in the resultant risk ratios.

**Table 4.** Poisson regression results, with risk ratios for COVID-19-related death in relation to daily corticosteroid dosage^a^

Daily steroid dose	Number of people	Number of COVID-19-related deaths	Risk ratio, unadjusted (95% CI)	*P*-value	Risk ratio, adjusted for age and sex (95% CI)	*P*-value
0 mg	8418	935	1		1	
>0 mg–5 mg	232	44	1.96 (1.43, 2.61)	**<0.01**	1.21 (0.88, 1.62)	0.21
>5 mg–10 mg	514	116	2.30 (1.89, 2.78)	**<0.01**	1.57 (1.29, 1.89)	**<0.01**
>10 mg–15 mg	259	54	2.09 (1.57, 2.72)	**<0.01**	1.46 (1.09, 1.89)	**<0.01**
>15 mg	585	142	2.49 (2.07, 2.95)	**<0.01**	2.15 (1.80, 2.56)	**<0.01**
Daily steroid dose, increasing in 5 mg increments	10 008	1291	1.11 (1.09, 1.13)	**<0.01**	1.10 (1.08, 1.13)	**<0.01**

a Participants whose positive COVID-19 PCR test result was received after death due to laboratory delays are included with a person-time of 10 days for this analysis, in keeping with the median time between positive test and death related by COVID-19 described by the WHO. Values in bold indicate *P*-value for trend of adjusted risk ratios is <0.01.

### Hospital and intensive care unit admissions

Demographic data for those who were admitted to hospital and/or to the ICU between 1 August 2020 and 30 April 2021 with a diagnostic code for COVID-19 are shown in Supplementary Table S2, available at *Rheumatology* online. The median age of those with a hospital admission was 73.6 years (IQR 59.6–82.5), and for an ICU admission 60.5 years (IQR 48.2–69.3). The median length of stay was 10 days (mean 15.7, range 0–269) and the median number of admissions was 2 (mean 2.5, range 1–23).

For ICU admissions, the median length of stay was 7 days (mean 12.7, range 0–170) and the median number of admissions was 1 (mean 1.3, range 1–5). The median number of basic respiratory support days while on ICU was 3 (mean 4.6, range 0–65) and the median number of advanced respiratory support days was 0 (mean 7.6, range 0–140) (Supplementary Table S5, available at *Rheumatology* online).

## Discussion

### Main findings

In the second wave of the COVID-19 pandemic, COVID-19-related death rates among people with RAIRD remained more than twice that of the general population. There was no significant difference between different RAIRDs.

Corticosteroid usage within the 30 days prior to COVID-19 infection was associated with an increased risk of COVID-19-related death, in a dose-dependent fashion. Patient education on this risk, and where appropriate steroid minimization strategies, should be considered.

### Strengths

This work supports our findings in the first wave. It also evaluates the influence of corticosteroids, a common cause for immunosuppression in people with RAIRD, on COVID-19 outcomes. This is the first time that this has been analysed in whole population data for people with RAIRD. This study also includes the novel use of community prescription data for rare disease analyses within the National Disease Registration Service.

Like our first study, a major strength of this work is that the denominator population is known, allowing us to describe rates of COVID-19 infection and of COVID-19-related death.

Our sub-analysis by disease continues to support the grouping together of RAIRD when assessing COVID-19 outcomes, to increase statistical power. The diseases have similar underlying disease mechanisms and immunosuppressive treatments and the risk of death related to COVID-19 is shown to be comparable between diseases. Unlike in the first wave, GCA showed the same risk profile as the other RAIRD, which may be a result of the narrower confidence intervals due to the increased number of events compared with the first wave.

### Limitations

The two measures of COVID-19 mortality described were selected to allow comparison with available statistics for the general population and with our earlier study [7]. We discussed in detail the drawbacks and merits of these measures in our earlier paper [7]. There is some evidence that people with RAIRD may have later COVID-19-related mortality than that of the general population, making the death within 28 days measure less suitable for this cohort [26].

As we identified our cohort from diagnoses in HES admitted patient care (APC) data, our methodology would not have identified patients treated entirely on an outpatient basis, who have never had an in-patient or daycase admission for any reason. Due to the complex nature of RAIRD, we believe this to be the minority of cases, and this is supported by the disease prevalence in our cohort being similar to previous studies [27–29]. However, this could skew our cohort towards those with more severe disease.

This study does not include data on immunosuppressive medications other than corticosteroids. People taking corticosteroids for their RAIRD may be on other immunosuppressants, which may compound their risk from COVID-19.

### Comparison to the published literature

Rare diseases are notoriously difficult to study as the small numbers of people affected by each condition reduces statistical power, making it harder to assess outcomes.

International studies describing COVID-19 in cohorts with AAV [2], GCA [3], Behçet’s disease [4] and systemic sclerosis (SSc) [5] demonstrated higher levels of COVID-19 infection when compared with the general population (in SSc this was only true of those with interstitial lung disease) but were limited by relatively small numbers [2, 3] and/or methods prone to inclusion bias [4], making it impossible to describe rates. Nationwide cohort studies from Denmark in people living with SLE [30] and vasculitis [31] showed an increased risk of hospitalization with COVID-19 but did not look at associated mortality.

Most COVID-19-related research has therefore grouped RAIRD with more common rheumatic diseases such as RA.

The COVID-19 Global Rheumatology Alliance described COVID-19-related mortality in rheumatic diseases, including RAIRD, and reported an association with immunosuppressive medication use [32]. Studies from the same group looking at individual conditions (idiopathic inflammatory myopathies (IIM) [10], SLE [33] and vasculitis combined with polymyalgia rheumatica [8]) described associations with steroid use, immunosuppressants including rituximab and COVID-19-related mortality. However, these studies relied on physician-reported cases and could not report rates of COVID-19 infection or deaths.

Whole population data from Denmark [34] found an increased risk of COVID-19 hospital admissions in people with connective tissue disease and vasculitis but did not report on COVID-19 infection rates, nor on mortality in RAIRD specifically. Whole population data from South Korea [35] also found an increased risk of COVID-19 infection and hospitalization. There was a suggestion of increased COVID-19 mortality but this was not statistically significant.

OpenSAFELY [36, 37] and QCOVID [38] looked at risk factors for severe COVID-19 outcomes in England, both combining SLE with other rheumatic diseases. Both found a modest increase in the risk of death (hazard ratio 1.32 [95% CI: 1.06, 1.65] and 1.20 [95% CI: 1.12, 1.28], respectively). A pre-print [39] from the QCOVID team looking at outcomes in the Omicron wave suggests a persistently increased risk.

At the time of the last published analyses, OpenSAFELY included 24 million patients [36, 37] and QCOVID 8.26 million [38]. Our research uses the whole England population of 56 million, affording us the statistical power to calculate more precise results. The use of whole population data, with a known denominator population, also allows calculation of rates and comparison to the general population.

### Infection rate

There was no significant difference in the age-standardized COVID-19 infection rate between people with RAIRD and the general population. This differs from the first wave, where the rate in people with RAIRD was around 50% higher.

This is likely to reflect limited testing availability early in the pandemic, where testing was predominantly in hospital with few tests available in the community. It may be that as people with RAIRD have increased contact with healthcare services, they underwent more frequent testing, leading to ascertainment bias. It could also reflect earlier vaccination in the RAIRD group due to prioritization of at-risk groups.

### Corticosteroids

COVID-19 registry studies in RAIRD have suggested an association with corticosteroid treatment and severe disease [9, 32, 40]. Adverse outcomes were associated with a prednisolone equivalent dose of 7.5–10 mg/day [1, 8, 10]. However, registry studies tend to include more serious COVID-19 cases due to selection bias, so it was unclear to what extent they reflected the wider risk. Additionally, many of these studies used combined outcomes (varyingly including hospitalization, oxygen supplementation, ventilation, death). Larger general population studies also showed an association between corticosteroid treatment and severe COVID-19 outcomes [38, 41].

Our study is the first specific to the RAIRD cohort to show an association between corticosteroid dose and COVID-19-related death and the first to be able to demonstrate a clear dose-dependent effect. It remains unclear whether this is a causative effect, or whether those on corticosteroid treatment are in poorer health with more active underlying disease, predisposing them to poorer outcomes.

Most corticosteroid prescriptions are issued in the community and so will have been captured in our data. While acute prescriptions issued in secondary care will not have been captured, this would lead us to underestimate total corticosteroid dose and therefore underestimate, rather than overestimate, the effect on COVID-19-related mortality.

### Clinical and policy implications and future research

People with RAIRD remained at increased risk of COVID-19-related death during the second COVID-19 wave, compared with people of the same age and sex in the general population. This has important implications for people living with RAIRD, their clinicians and for public health policy, to protect their health and reduce the risk of severe outcomes.

RAIRD may require immunosuppression with corticosteroids. We have demonstrated that corticosteroids are associated with poorer COVID-19 outcomes, and this should form part of the decision-making process when considering a corticosteroid prescription.

As during the first wave, we did not find an excess of non-COVID-19-related deaths. This likely reflects efforts across healthcare to prioritize access to emergency care. However, a delayed impact on mortality rates due to delayed or missed diagnoses, particularly from conditions such as cancer, remains possible.

## Supplementary Material

kead150_Supplementary_Data

## Data Availability

Due to legal and ethical considerations, supporting data cannot be made openly available. However, NCARDRS data are available to all who have a legal basis to access them. Further details about the data and conditions for access are available by application to the National Disease Registration Service (https://digital.nhs.uk/ndrs). Information on how to access this data can also be obtained from the University of Nottingham data repository: DOI: 10.17639/nott.7272.
