# Supplementary material for: COVID-19 infection, admission and death and the impact of corticosteroids among people with rare autoimmune rheumatic disease during the second wave of COVID-19 in England: results from the RECORDER Project
Source: Rheumatology (Oxford). 2023 Apr 5;62(12):3828–37. doi: 10.1093/rheumatology/kead150 (PMC10691923; doi:10.1093/rheumatology/kead150)
Supplement: kead150_Supplementary_Data [file kead150_supplementary_data.docx]

| Supplementary Table S1: Deaths and age-standardised mortality rates 01 August 2020 to 30 April 2021 for the RAIRD cohort, compared to the 2013 European Standard Population | | | | | | | |
| --- | --- | --- | --- | --- | --- | --- | --- |
|  | Number of deaths | Number of people | Person-years | Crude mortality rate per 100,000 person years | RAIRD age-standardised mortality rate | General population age-standardised mortality rate | Risk ratio for mortality rates |
| All-cause mortality | | | | | | |  |
| All | 5,822 | 168,330 | 125,902 | 4,611.6 (4509.0 – 4714.2) | 3064.6 (3006.9 – 3122.3) | 1,042.6 (1039.9 – 1045.3) | 2.94 (2.88 – 2.99) |
| Death with any mention of COVID-19 on the death certificate | | | | | | |  |
| All | 1,342 | 168,330 | 125,902 | 1063.0 (1013.7 – 1112.2) | 506.9 (483.4 -530.4) | 200.9 (199.7 – 202.1) | 2.52 (2.41 – 2.64) |
| Death within 28 days of a positive COVID-19 test | | | | | | | |
| All | 1,196 | 168,330 | 125,902 | 947.3 (900.8 – 993.8) | 451.9 (429.7-474.1) | 184.3 (183.2-185.5) | 2.45 (2.33 – 2.57) |
| **Note:** <1 and 1-4 age groups plus 90-94 and 95+ age groups were combined for calculation, to align with format of available general population denominator data. | | | | | | | |

| Supplementary Table S2: ONS ascribed underlying cause of death by category in RAIRD cohort between 01 August 2020 and 30 April 2021 | |
| --- | --- |
| Cause of death | n (% total deaths) |
| Category |  |
| Cardiovascular | 1,216 (21.5%) |
| COVID-19 | 1,194 (21.1%) |
| Malignancy | 1,020 (18.0%) |
| Other | 1,004 (17.8%) |
| Respiratory | 539 (9.5%) |
| Dementia | 430 (7.6%) |
| Underlying RAIRD | 193 (3.4%) |
| Non COVID-19 infection | 55 (1.0%) |
| Total deaths in RAIRD population with death certificate data available | 5,651 |
| Total RAIRD population | 168,330 |

| Supplementary Table S3: Age at death of RAIRD cohort August-April 2016-2021 | | |
| --- | --- | --- |
| Year | Median age | IQR |
| 2020-2021 |  |  |
| All deaths | 80.8 | 72.0-87.2 (15.2) |
| COVID-related | 80.2 | 70.8-86.6 (15.8) |
| Non-COVID-related | 81.0 | 72.2-87.5 (15.3) |
| 2019-2020 | 80.8 | 72.2-87.5 (15.3) |
| 2018-2019 | 80.8 | 71.6-87.3 (15.6) |
| 2017-2018 | 80.9 | 71.9-87.5 (15.6) |
| 2016-2017 | 80.7 | 71.4-87.3 (15.9) |

| Supplementary Table S4: Poisson regression results with risk ratios for COVID-19-related death in relation to daily corticosteroid dosage, including those with zero person-time† | | | | | | |
| --- | --- | --- | --- | --- | --- | --- |
| Daily steroid dose | Number of people | Number of COVID-19 related deaths | Risk ratio – unadjusted (95% CI) | p-value | Risk ratio – adjusted for age and sex (95% CI) | p-value |
| 0mg | 8,435 | 949 | 1 |  | 1 |  |
| >0mg-5mg | 235 | 47 | 2.06 (1.52 – 2.73) | <0.01* | 1.28 (0.94 – 1.69) | 0.1 |
| >5mg-10mg | 518 | 120 | 2.35 (1.93 – 2.82) | <0.01* | 1.60 (1.32 – 1.93) | <0.01* |
| >10mg-15mg | 259 | 54 | 2.06 (1.55 – 2.68) | <0.01* | 1.43 (1.08 – 1.87) | 0.01* |
| >15mg | 587 | 144 | 2.48 (2.08 – 2.95) | <0.01* | 2.15 (1.80 – 2.56) | <0.01* |
| Daily steroid dose, increasing in 5mg increments | 10,034 | 1,314 | 1.11 (1.09 – 1.13) | <0.01* | 1.10 (1.08 - 1.13) | <0.01* |
| †In this sub-analysis, those with zero person-time are included using 0.5 days person-time. 28 people whose positive PCR tests returned after death due to laboratory delays are excluded. | | | | | | |

| Supplementary Table S5: Summary of hospital and intensive care unit (ICU) admissions, including demographics and characteristics of stay | | | | |
| --- | --- | --- | --- | --- |
| Demographics | | | | |
|  | n | Mean age | Median age | IQR |
| Any admission with COVID code | 4,433* | 69.6 | 73.6 | 59.6-82.5 (22.9) |
| Death certificate mention of COVID-19 | 1,081† | 76.6 | 78.9 | 69.9-85.2 (15.3) |
| Death within 28 days of positive COVID-19 PCR | 958 | 76.6 | 78.7 | 70.2-85.1 (14.9) |
| ICU admission with COVID code | 387 | 58.6 | 60.5 | 48.2-69.3 (21.1) |
| Death certificate mention of COVID-19 | 128 | 64.7 | 65.2 | 57.8-74.5 (16.7) |
| Death within 28 days of test | 155 | 64.3 | 65.4 | 57.3-75.4 (18.2) |
| COVID positive, not admitted, all | 6,947 | 49.1 | 49.3 | 29.9-65.8 (35.9) |
| Death from all causes | 315 | 81.0 | 84.3 | 76.1-89.6 (13.5) |
| Death certificate mention of COVID-19 | 261 | 81.6 | 84.6 | 76.1-89.9 (13.8) |
| Death within 28 days of test | 240^§^ | 81.1 | 84.4 | 75.7-89.8 (14.1) |
| Mention of COVID-19 on death certificate, without admission or positive PCR | 36 | 82.6 | 84.6 | 73.8-90.6 (16.8) |
| *Of whom 3014/4433 had a positive COVID-19 PCR test  † Of whom 1015/1081 had a positive COVID-19 PCR test  ^§^Of whom 211/240 had mention of COVID-19 on their death certificate | | | | |
| All hospital admissions with a diagnostic code for COVID-19‡ (n=4432) | | | | |
|  | Median | Mean | Range | IQR |
| Duration of admission (days) | 10.0 | 15.7 | 0.0-269.0 | 4.0-21.0 |
| Number of admissions per individual | 2.0 | 2.5 | 1.0-23.0 | 1.0-3.0 |
| ICU admissions with a diagnostic code for COVID-19‡ (n=387) | | | | |
|  | Median | Mean | Range | IQR |
| Basic respiratory support days | 3.0 | 4.6 | 0.0-65.0 | 1.0-6.0 |
| Advanced respiratory support days | 0.0 | 7.6 | 0.0-140.0 | 0.0-8.0 |
| Duration of admission (days) | 7.0 | 12.7 | 0.0-170.0 | 3.0-15.0 |
| Number of ICU admissions per individual | 1.0 | 1.3 | 1.0-5.0 | 1.0-1.0 |
| ‡Where an individual had more than one admission, totals are summed | | | | |

Supplementary Figure S1: Deaths in RAIRD cohort between 01 August 2020 and 30 April 2021, shown as all deaths, deaths with any mention of COVID-19 on the death certificate and deaths within 28-days of a positive COVID-19 PCR test, with all deaths in RAIRD cohort between 01 August 2019 and 30 April 2020 as a comparator
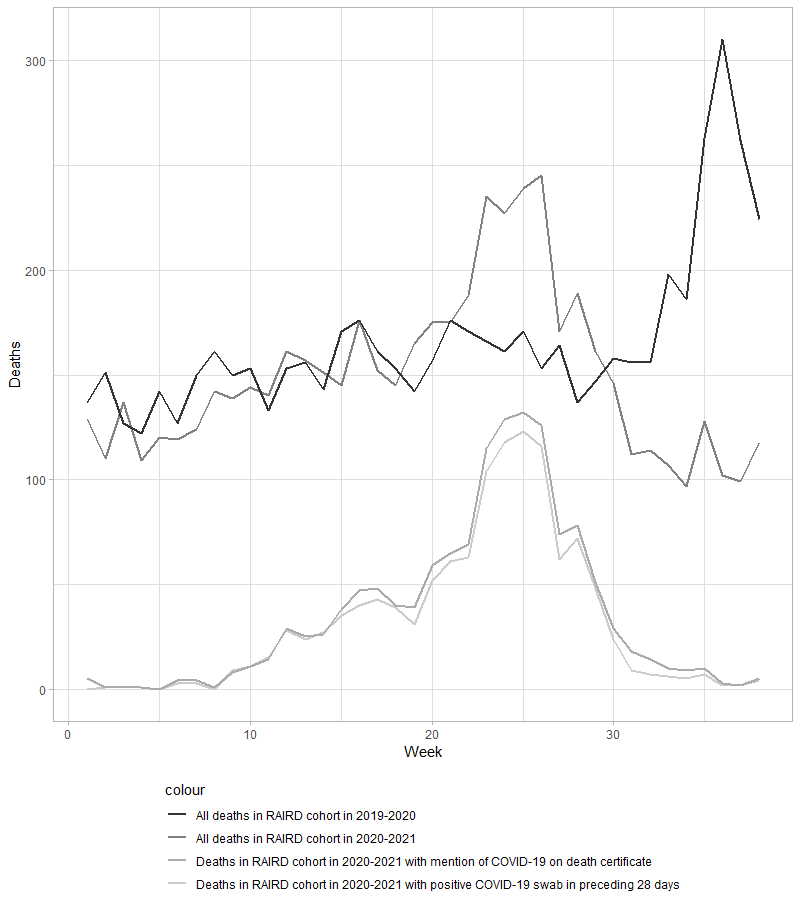


Supplementary Figure S2: Cause of death by category and age in people with RAIRD i) between August 2020 to April 2021 and ii) mean over August to April 2016-2020

1. **
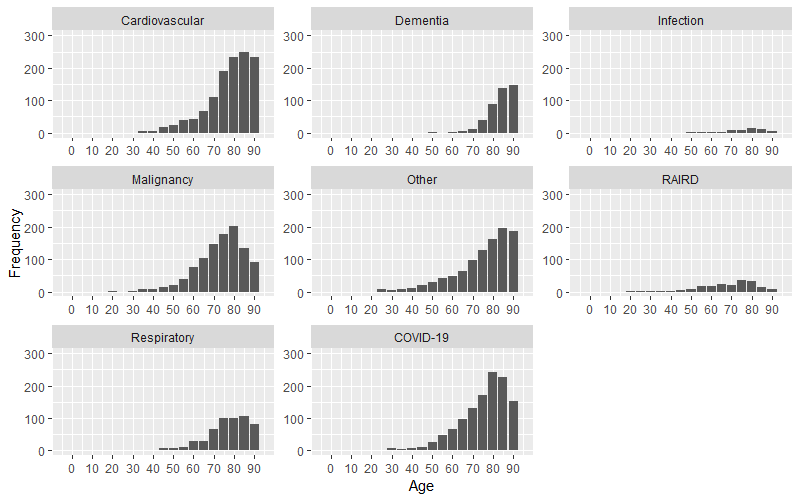
**

ii)

**
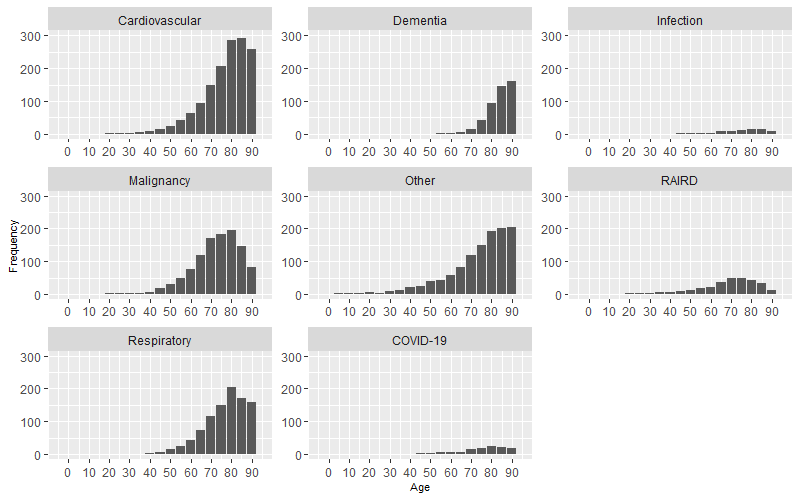
**

Supplementary Data S1:

Algorithm for assigning main rheumatological diagnosis

All records with only non-specific CTD codes remaining removed

Where primary diagnosis was a non-specific CTD code

(“Glomerular disorder in systemic connective tissue disorder” (N08.5),

“Renal tubulo-interstitial disorder in systemic connective tissue disorder” (N16.4), “Respiratory disorder in other diffuse connective tissue disorder” (J99.1))

Replaced with most recent specific diagnostic code

Most recent diagnostic code for RAIRD applied as primary RAIRD diagnosis

Process repeated 8 times (until no further diagnostic codes)

Process repeated 8 times (until no further diagnostic codes)

Where primary diagnosis was “Polyarteritis Nodosa” or “Arteritis, unspecified” (I776), replaced with most recent specific diagnostic code (process not applied where next most recent code was a non-specific CTD code)

Supplementary Data S2:

The combined total of people with RAIRD dying with either COVID-19 mentioned on their death certificate, or within 28 days of a positive COVID-19 test, was 1,415/168,330 (0.84%) and by this measure COVID-19 was implicated in 1,415/5,822 (24.3%) of all deaths during this time-period in this cohort. There is no similar data for the general population of England with which to compare this.
